# Venous Thromboembolic Events in Cancer Immunotherapy: A Narrative Review

**DOI:** 10.3390/jcm14144926

**Published:** 2025-07-11

**Authors:** Cosmo Fowler, Stephen M. Pastores

**Affiliations:** 1 Division of Pulmonary, Allergy, Critical Care, and Sleep Medicine, Department of Medicine, Emory University School of Medicine, Atlanta, GA 30322, USA; 2 Critical Care Center, Department of Anesthesiology and Critical Care Medicine, Memorial Sloan Kettering Cancer Center, New York, NY 10065, USA; 3 Departments of Anesthesiology and Medicine, Weill Cornell Medical College, New York, NY 10021, USA

**Keywords:** cancer, immunotherapy, thrombosis, venous thromboembolism, pulmonary embolism, deep vein thrombosis, cancer-associated thromboembolism, immune checkpoint inhibitor, chimeric antigen receptor T-cell therapy, cellular therapy

## Abstract

Venous thromboembolism (VTE) represents a significant complication of cancer immunotherapy, with emerging evidence suggesting distinct pathophysiological mechanisms compared to traditional chemotherapy-associated thrombosis. This narrative review examines the epidemiology and pathogenesis of VTE in patients receiving immunotherapies for cancer including immune checkpoint inhibitors (ICIs), chimeric antigen receptor (CAR) T-cell therapy, bispecific T-cell engagers (BiTEs), among others. Real-world studies demonstrate a wide range of VTE incidence rates in ICI recipients, with potential mechanisms including exacerbated underlying interleukin-8-mediated inflammatory pathways and consequent neutrophil extracellular trap (NET) formation. CAR T-cell therapy is associated with unique hemostatic challenges, including concurrent thrombotic and bleeding risks related to cytokine release syndrome. Current risk assessment tools show limited predictive utility in patients receiving immunotherapies for cancer, highlighting the need for novel stratification models. Future research priorities include developing immunotherapy-specific risk prediction tools, elucidating mechanistic pathways linking immune activation to thrombosis, and establishing evidence-based and tailored thromboprophylaxis strategies. As cancer immunotherapy continues to evolve, understanding and mitigating thrombotic complications remains crucial for optimizing patient outcomes.

## 1. Introduction

Cancer has long been recognized as a prothrombotic state, with malignancy conferring a substantially elevated risk of thromboembolism [1,2,3,4,5,6]. Venous thromboembolic events (VTEs), which comprise both deep vein thrombosis (DVT) and pulmonary embolism (PE), are a significant source of morbidity in patients with cancer and a leading cause of death in this population [7], second only to the cancer itself [8,9]. This elevated thrombotic risk is multifactorial, involving tumor biology, patient-specific factors, and treatment-related elements [2,6].

In 1868, Wilhelm Busch published observations on the regression of sarcoma of the neck brought about by deliberate infection with *Streptococcus pyogenes* [10]. This approach, later disseminated by William H Coley after performing similar experiments at New York’s Memorial Hospital in the 1890s, represents one of the earliest-studied forms of immunotherapy [10]. In the late 20th century, cytokines were trialed and then approved by the Food and Drug Administration (FDA) for hematologic malignancy and in 1997, the CD20-targeting rituximab became the first monoclonal antibody approved for the treatment of cancer [11].

It was in 2011, with the FDA’s approval of ipilimumab, a cytotoxic T-lymphocyte associated protein 4 (CTLA-4)-targeting immune checkpoint inhibitor (ICI) for the treatment of melanoma that cancer immunotherapy entered a renaissance. Some 150 years after the publication of Busch’s accounts, this advance ushered in an explosion of ICI therapies, now numbering over a dozen for well over a hundred indications [12]. Alongside these, cellular immunotherapies such as chimeric antigen receptor (CAR) and T-cell receptor (TCR) T-cells, cancer vaccines, and oncolytic viral therapies continue to be carried from bench to bedside [10,11,13].

With the advent of ICI therapy, a new spectrum of toxicities emerged, termed immune-related adverse events (irAEs). These irAEs are autoimmune conditions which can affect virtually any organ system through immune checkpoint blockade, and can differ in pathophysiology, onset latency, and acuity. In parallel, adoptive cell therapies, also known as “living drugs”, are also associated with novel, cytokine-mediated toxicities. CAR T-cell therapy in particular is known to induce multisystemic, inflammatory responses such as cytokine release syndrome (CRS) and immune effector cell-associated hemophagocytic lymphohistiocytosis-like syndrome (IEC-HS) [14,15,16].

While there is relatively little described on the association between many of these novel cancer immunotherapies and VTE risk, some, such as ICIs, have been linked to overall rates of thromboembolism as high as 24% [17]. This presents a significant clinical challenge, as the eligibility of patients with advanced or metastatic cancer for ICIs alone has increased from just over 1% in 2011 to over 50% in 2023 [18]. Here, we review and summarize recent and landmark findings in the oncological literature on the incidence and pathogenesis of VTE in cancer patients receiving immunotherapy, as well as its diagnosis, management, and prevention.

## 2. Cancer-Associated Thromboembolism

### 2.1. Epidemiology

Cancer accounts for 20–30% of all initial VTE episodes, with patients with cancer having a 4–7 times higher risk than non-cancer patients [19]. Cancer is, in and of itself, one of the strongest identified risk factors for VTE occurrence: in one population study conducted in Australia, cancer conferred a relative risk (RR) of VTE of approximately 15 [20], an effect that was even more marked among younger patients. In those patients, the risk was magnified over a hundredfold, with an RR of up to 139.3 (95% CI 78.6–246.7) [20].

The incidence of cancer-associated thromboembolism (CAT), both venous (VTE) and arterial thromboembolism, has increased steadily over the past two decades [1,21,22,23]. A survey of over 3 million cancer patients hospitalized in U.S. medical centers observed rates of CAT almost double between 1995 and 2012 (3.5% vs. 6.5%) [23]. One large population-based Danish study found the 12-month VTE incidence of 3.4% in 2017 more than tripled among patients with a first-time cancer diagnosis, up from 1% in 1997 [21]. While these changes are multifaceted and reflect in part improvements to surveillance strategies, the shifting therapeutic landscape for cancer is just as inextricable [1]. An earlier, United Kingdom-based study of patients with active cancer recorded between 2001 and 2011 reported an incidence rate of VTE of 5.8 per 100 person-years (approximating a 12-month cumulative incidence of 5.6%) [24]. This study also described a 12-month mortality rate 64.5% among patients with CAT; this resembled the 12-month cumulative mortality reported by a separate Danish study (68.2%) [25]. Finally, while mortality among U.S. inpatients with CAT dropped from 18.1% in 1995 to 12.9% in 2012, it remained three times higher than patients without a VTE diagnosis (4.3%) [23]. It should be noted that while the 12-month mortality rate among patients with CAT remains substantial, this elevated mortality reflects the complex interplay between cancer progression and thrombotic complications rather than VTE as an isolated cause of death.

In patients receiving chemotherapy, real-world analyses report 12-month cumulative VTE incidences ranging 9.8% to 21.3%, with substantial variation by cancer type, stage, and treatment regimen [26]. While these changes reflect improvements in surveillance strategies and diagnostic imaging sensitivity, they also mirror the shifting therapeutic landscape, including more intensive combination chemotherapies. Certain regimens of chemotherapy may amplify thrombotic risk substantially, with cisplatin conferring particularly high risk (adjusted RR 3.3, 95% CI 1.6–6.8) [27]. A retrospective study of over 900 patients treated with cisplatin-based chemotherapy found that the vast majority (88%) of cases of CAT occurred within 100 days of treatment initiation (median time to thromboembolism of 48 days, IQR 26–73) [28].

### 2.2. Pathophysiology

The pathophysiology of CAT involves profound alterations in hemostasis affecting each element of Virchow’s triad, including altered blood flow (stasis), endothelial dysfunction, and hypercoagulability [1,3,19,29]. This prothrombotic transformation involves direct tumor cell effects, host–tumor interactions, and systemic inflammatory responses that collectively create an environment primed for thrombosis.

#### 2.2.1. Tissue Factor Expression and Direct Procoagulant Activity

Tissue factor (TF) expression plays a critical role in cancer-associated coagulopathy [1,30]. Not only is TF often expressed on cancer cells and the tumor vasculature, TF-bearing microparticles can be shed by these cells and have been identified as a key mediator in the development of CAT [30,31,32]. Moreover, elevated levels of circulating TF-positive extracellular vesicles have been identified clinically in patients with numerous cancer types, and in the case of pancreatic cancer, have been significantly associated with the development of CAT [1,31].

Beyond membrane- and microparticle-bound TF, cancer cells express other procoagulants, such as podoplanin. Podoplanin has been identified on several tumor cells and induces platelet aggregation as a ligand of platelet receptor C-type lectin receptor type-2 [1,2,19,30]. Podoplanin and other procoagulants, such as polyphosphate, may be released by tumor cells in extracellular vesicles, as in the case of TF [1]. Additional molecules, such as cancer procoagulant, which directly activates clotting factor X independently of factor VII, heparinase, and plasminogen activator inhibitor 1, have all been described across multiple tumor cells and contribute to a hypercoagulable phenotype [1,2,19,30].

#### 2.2.2. Endothelial Dysfunction in Cancer

Beyond endothelial injury caused by direct tumoral invasion or secondary to therapeutic approaches (e.g., catheter placement), the tumor microenvironment profoundly disrupts normal endothelial homeostasis, shifting the vascular lining from an anticoagulant to a prothrombotic surface [1,19]. Cancer-induced inflammatory cytokines, particularly tumor necrosis factor-α (TNF-α) and IL-1β, downregulate endothelial thrombomodulin expression 70–90% through NF-κB-mediated transcriptional repression, while simultaneously reducing protein C receptor expression and upregulating endothelial TF expression [33,34,35]. Tumor cells may also trigger the release of von Willebrand factor from Weibel–Palade bodies in the endothelium, promoting platelet adhesion [36]. Additionally, the tumor microenvironment’s hypoxic conditions result in activation of hypoxia inducible factor 1α, which further upregulates TF expression in both tumor and stromal cells while promoting vascular endothelial growth factor-mediated angiogenesis, compounding endothelial dysfunction [37].

#### 2.2.3. Cytokine-Driven Thrombogenesis

The inflammatory milieu of cancer contributes to thrombogenesis through specific cytokine networks that link inflammation and coagulation. Three cytokines, IL-6, TNF-α, and IL-1β, serve as central mediators of cancer-associated hypercoagulability through distinct but overlapping mechanisms [30,38]. IL-6, markedly elevated in cancer patients and with levels correlating with disease stage and prognosis [39,40], exerts profound prothrombotic effects by inducing hepatic synthesis of fibrinogen and factor VIII, upregulating TF on monocytes, while suppressing anticoagulant protein S and antithrombin [38,41]. TNF-α promotes thrombosis by inducing endothelial TF expression, increasing monocyte chemoattractant protein-1, and synergizing with IL-1β to upregulate plasminogen activator inhibitor-1, creating a sustained prothrombotic endothelial phenotype [41].

#### 2.2.4. Chemotherapy-Associated Thromboembolism

As discussed, chemotherapy substantially amplifies baseline thrombotic risk in cancer patients through both drug-specific and class-effect mechanisms. Chemotherapy induces direct endothelial toxicity, evidenced by rapid von Willebrand factor released from damaged endothelium, with cisplatin-based regimens showing particularly early vascular injury [42]. Multiple chemotherapeutic agents decrease natural anticoagulant levels including protein C and protein S, while simultaneously increasing TF expression [43]. Newer targeted therapies present distinct thrombotic mechanisms, such as the anti-angiogenic agent bevacizumab and multi-kinase inhibitors, which disrupt vascular integrity, reduce nitric oxide production and promote endothelial dysfunction [2,19,44].

In summary, CAT arises from the convergence of multiple prothrombotic mechanisms inherent to malignancy, modulated by host factors, and amplified by therapeutic interventions. Understanding of these complex interactions has enabled risk stratification approaches and targeted prevention strategies, though the optimal balance between thrombosis prevention and bleeding risk remains an ongoing clinical challenge.

## 3. Immune Checkpoint Inhibitor Therapy

### 3.1. Epidemiology

Multiple investigators have described an association between ICI therapy and VTE. In one of the largest single-center studies published to date, Gong et al. described a 1-year VTE incidence of 13.8% after starting ICI therapy, with a circa 5-fold increase (hazard ratio [HR] of 4.98) in VTE risk after therapy initiation [45]. The most commonly represented cancer types in this cohort were non-small cell lung cancer and melanoma (28.4% and 28.2%, respectively), and the majority (75.2%) of patients were prescribed programmed cell death protein 1 (PD-1)-targeting agents. Another large, single-center retrospective cohort study in the US (1686 ICI recipients, 49.6% lung and 13.2% melanoma) reported a crude VTE proportion of 24% among patients receiving ICIs, with a 12-month cumulative incidence of 10.9% [17]. Most of the cohort received PD-1 targeting agents (51.5% nivolumab and 27.1% pembrolizumab), and the distribution of ICIs was not significantly different between VTE and non-VTE cases [17] Further, VTE was associated with an overall decrease in survival with a HR of 1.22 (95% CI 1.06–1.41) [17]. An earlier retrospective cohort of 672 Viennese ICI recipients (30.4% melanoma, 24.1% lung) reported a cumulative VTE incidence of 12.9% (median follow-up 8.5 months) [46].

While several other studies have detailed the elevated risk of CAT in ICI recipients [47,48,49], there are conflicting data informing how this risk compares to similar patients treated with systemic chemotherapy—in part, this is because in many circumstances, ICI therapy has become the standard of care [17]. In a retrospective study of the U.S. Veteran Affairs Cancer Registry, the authors used propensity score matching to compare 1823 advanced stage cancer patients treated with ICIs to 6345 similar patients treated with cytotoxic chemotherapy; 6-month CAT incidence was 8.36% vs. 8.49%, respectively (adjusted HR 1.06, 95% 0.88–1.26) [50]. Meta-analyses of randomized controlled trials (RCTs) between ICI and non-ICI regimens have identified similar risks of VTE [51]. One of these meta-analyses also reported on a significantly higher risk of arterial thromboembolic events among ICI recipients, as a higher risk of PE among lung cancer patients elicited in a sub-analysis stratifying by cancer type [51]. Finally, in one large database study examining thromboembolic risk in platinum combination chemotherapy recipients for non-small cell lung cancer, the addition of ICI resulted in a higher risk of VTE (HR 1.27, 95% CI 1.01–1.60) [52].

### 3.2. Pathophysiology

The pathophysiology of ICI-associated thrombosis represents a complex interplay of immune activation, inflammatory cascades, and hemostatic dysregulation distinct from traditional chemotherapy-induced coagulopathy. Unlike chemotherapy, ICI-associated thrombosis stems fundamentally from immune-mediated mechanisms, which may contribute to the distinct temporal trends of its incidence, as described above [53]. Recent mechanistic studies have identified multiple interconnected pathways that collectively create a unique prothrombotic state in ICI recipients.

#### 3.2.1. Inflammatory Cascade

ICI therapy transforms the tumor microenvironment’s inflammatory landscape, creating a prothrombotic state that extends systemically through multiple interconnected pathways. Checkpoint blockade may result in extensive remodeling of the tumor microenvironment, characterized by enhanced T cell infiltration, cytokine release, and altered cellular composition [54]. Central to this process is interferon-γ (IFN-γ), whose levels increase over 2-fold in both tumor and plasma following ICI administration, with a strong correlation between compartments [55]. IFN-γ serves as a master regulator of ICI-associated coagulopathy, directly upregulating TF expression on tumor cells via JAK1/2-STAT1 signaling and promoting the release of TF-positive extracellular vesicles through the IRF1-Rab27a axis [55]. The IFN-γ-TF axis is well-established, with various inflammatory cytokines shown to induce TF expression in various cell types, such as endothelial cells [55,56,57]. Additionally, elevated levels of soluble vascular cell adhesion molecule 1 (sVCAM-1, which may be derived from activated endothelial cells) observed in ICI recipients who develop thrombosis suggest that systemic endothelial activation may also be present in these individuals [9,17]. Furthermore, elevated levels of inflammatory cytokines including IL-6 and IL-1β increase blood viscoelasticity, and the combination of these cytokines can induce platelet activation and spreading [17,58].

#### 3.2.2. PD-1/PD-L1 Effects on Platelet Function

Beyond T-cell modulation, the PD-1/PD-L1 axis directly affects platelet biology. Platelets express PD-L1 on their surface and within α-granules, with expression significantly increased in cancer patients [59]. This platelet PD-L1 regulates activation through the Caspase-3/GSDME pathway, promoting IL-1β release and integrin αIIbβ3 outside-in signaling that enhances platelet aggregation and spreading [60]. Additionally, platelets acquire PD-L1 from tumor cells through fibronectin 1, integrin α5β1, and GPIbα-dependent mechanisms, creating “tumor-educated” platelets with enhanced prothrombotic potential [61].

#### 3.2.3. MDSC Recruitment and NET Formation

Roopkumar et al. identified a potential IL-8-mediated pathway that may contribute to immunotherapy-associated thrombosis [9,17]. Their study demonstrated that patients who developed VTE following ICI therapy had significantly higher pretreatment levels of IL-8, myeloid-derived suppressor cells (MDSCs), and sVCAM-1 compared to patients who did not develop CAT [9,17]. The authors proposed that ICIs may therefore worsen preexisting cancer-associated inflammation, with IL-8 released by tumor cells and other cells in the tumor microenvironment promoting MDSC accumulation [9,17]. Through activation of CXCR1/2, IL-8 induces the release of neutrophil extracellular traps (NETs) by tumor-infiltrating MDSCs. NETs play a critical role in thromboinflammatory disorders, serving as scaffolds for thrombus formation through multiple mechanisms including complement activation, contact pathway activation and direct platelet activation via histone-Toll-like receptor 2/4 interactions [3,4,17,41]. Citrullinated histone H3, a NET biomarker, predicted VTE risk with an HR of 1.8 (95% CI 1.2–2.7), supporting the clinical relevance of this pathway [62].

## 4. Adoptive Cell Therapy

### 4.1. Epidemiology

The landscape of adoptive cell therapy has rapidly evolved beyond conventional CAR T-cell approaches, with emerging platforms including TCR-engineered T cells, tumor infiltrating lymphocytes (TILs), CAR NK cells, and other engineered cellular products. Understanding the thrombotic risk profile across these diverse therapeutic modalities remains challenging due to heterogenous patient populations, varying disease states, and inconsistent adverse event reporting.

#### 4.1.1. CAR T-Cell Therapy

Now with seven FDA-approved agents, CAR T-cell therapies represent the most widely-adopted form of adoptive cell therapy and are indicated for the treatment of relapsed or refractory hematologic malignancies including large B-cell lymphoma (LBCL) and ALL, among others [63]. CARs are generated through a process of host T-cell apheresis, genetic engineering to express a tumor antigen-specific receptor, growth, and re-infusion [64]. A recent systematic review and meta-analysis described a pooled VTE incidence of 2.4% per patient-month among CAR T-cell therapy recipients, comparable to that of high-risk cancer patients treated with cytotoxic chemotherapy [64]. This analysis, primarily derived from studies of axicabtagene ciloleucel (also known as axi-cel), a CD19-targeting CAR used in relapsed/refractory LBCL, likely underestimates the true incidence given retrospective data collection and variable follow-up periods [65,66,67].

#### 4.1.2. TCR T-Cell Therapy

TCR T-cell therapies are developed through a process similar to that employed in CAR T-cell generation, except that instead of a CAR, host T-cells are engineered to express natural TCRs able to target multiple intracellular tumor antigens presented by major histocompatibility complex cellular surface proteins. Afamitresgene autoleucel, or afami-cel, was approved by the FDA in 2024 for metastatic synovial sarcoma following a phase 2 international open-label trial (SPEARHEAD-1), and remains at the time of this writing the only approved TCR therapy [63,68]. SPEARHEAD-1 included 52 patients from across 23 institutions, and reported one instance each of DVT and PE (3.8% combined incidence) [68]. However, longer follow-up and larger patient cohorts are needed to establish the true thrombotic risk, particularly given the solid tumor indication which may carry significantly different baseline risks from hematologic malignancies.

#### 4.1.3. Tumor-Infiltrating Lymphocyte Therapy

TILs are a form of adoptive cell therapy in which natural T cells are grown ex vivo from resected tumor tissue and then reinfused, taking advantage of a host of natural TCRs targeting numerous tumor antigens [63]. Lifileucel was granted accelerated approval for metastatic melanoma by the FDA in 2024, and while the literature specifically assessing the association of this therapy with VTE is lacking, a phase 3 trial in which 84 patients were assigned to receive TIL therapy for melanoma reported two instances of PE (2.5%, vs. 1.2% in the ipilimumab treatment arm) [69]. Of note also, an integral component of this therapy is high-dose interleukin (IL)-2, on its own an early form of cytokine immunotherapy first approved in 1992 for metastatic renal cancer, used here to facilitate TIL expansion [63,69]. While some authors have raised concerns about adverse thromboembolic events in IL-2 therapy trials (with one important example being the ESPRIT study, conducted in human immunodeficiency virus [HIV]-infected patients) [70], a 2019 meta-analysis of RCTs involving IL-2 found a pooled risk difference for thromboembolic complications of approximately 0% [71].

#### 4.1.4. CAR NK Cell Therapy

CAR NK cells (natural killer cells engineered with chimeric antigen receptors) represent an emerging platform with potential advantages over CAR T-cells, including reduced CRS severity and off-the-shelf availability [72,73,74,75]. Early clinical data from trials of CD19-directed CAR NK cells in B-cell malignancies suggest a favorable safety profile with minimal CRS [73,74,75]. Among 11 patients treated in the initial phase 1/2 trial, no thrombotic events were reported despite 73% achieving remission, and another phase 1/2 trial of 37 patients did not report any instances of VTE [74,75].

### 4.2. Pathophysiology

#### 4.2.1. CAR T-Cell Therapy

CAR T-cell therapy induces thrombotic complications through mechanisms that differ fundamentally from conventional chemotherapy-associated thrombosis, with coagulopathy appearing closely linked to CRS development [63,64,76,77]. Following CAR T-cell activation and expansion, the release of pro-inflammatory cytokines creates a hypercoagulable state through multiple interconnected pathways that affect virtually every component of the hemostatic system.

Endothelial activation represents a central mechanism in CAR T-cell-associated coagulopathy [78,79]. Monocyte-derived IL-1 initiates the cascade, preceding IL-6 by approximately 24 h [79]. This process then disrupts the angiopoietin-TIE2 (angiopoietin’s receptor) axis, with markedly elevated ANG-2:ANG-1 ratios indicating widespread endothelial activation [14,77,78]. Activated endothelial cells release von Willebrand factor and lead to a prothrombotic state, one characterized by consumptive coagulopathy, capillary leak syndrome, and increased blood–brain barrier permeability [14,77,78]. Inflammatory cytokines, particularly IFN-γ, induce brain pericyte stress and secretion of IL-6, amplifying permeability which can in severe cases culminate with widespread vascular disruption, microhemorrhages and intravascular microthrombi [14,77,78].

In IEC-HS, which comprises a hyperinflammatory syndrome beyond conventional CRS characterized by macrophage activation, persistent fever, and multiorgan dysfunction, disseminated intravascular coagulation has been described [14,15,16]. The pathophysiology involves excessive macrophage activation with hemophagocytosis, leading to consumptive coagulopathy more severe than in isolated CRS [14,16]. Endothelial activation remains central, with disruption of the angiopoietin-TIE2 axis and markedly elevated ferritin levels [14,16].

#### 4.2.2. TCR T-Cell Therapy

TCR T-cell therapies undergo a manufacturing process similar to CAR T-cell generation, though possess distinct antigen recognition mechanisms, with TCR T cells targeting intracellular tumor antigens presented by major histocompatibility complex proteins rather than surface antigens [63,80]. Clinical experience with TCR T-cell therapies demonstrates rates of CRS occurrence similar to CAR T-cells (40–70% of patients treated with NY-ESO-1 or MAGE-A4 TCR T-cells) [81,82]. The cytokine profile includes elevation of IL-6, IFN-γ, TNF-α, and IL-2, all of which contribute to a prothrombotic milieu through endothelial activation [83].

#### 4.2.3. Tumor-Infiltrating Lymphocyte Therapy

TIL therapy presents unique thrombotic considerations due to its integral use of high-dose IL-2, which has well-established effects on vascular integrity and coagulation. IL-2 primarily disrupts endothelial barrier function through VE-cadherin redistribution and endocytosis, and while it does not directly induce TF expression, it stimulates the production of secondary cytokines among endothelial cells, including IL-1 and TNF-α, in this way facilitating a procoagulant phenotype [66,67]. The expanded TIL population itself also contributes to coagulopathy through cytokine release, including IFN-γ and TNF-α [68].

#### 4.2.4. CAR NK Cell Therapy

CAR NK cell therapy demonstrates a substantially reduced toxicity profile compared to CAR T-cell therapy, with lower rates of CRS and neurotoxicity across numerous trials [73,74,75]. CAR NK therapy also exhibits a distinct cytokine profile compared to CAR T-cell therapy, with one trial showing only modest elevations in IL-6 and IL-1β levels, which appear to be key drivers in CRS-associated coagulopathy [75]. Despite the theoretical risk of CAR NK-triggered cytokine release inducing a prothrombotic state, this has not yet materialized in available trial data [74,75].

## 5. Bispecific T-Cell Engager Therapy

### 5.1. Epidemiology

Bispecific T-cell engagers (BiTE) are monoclonal antibodies that simultaneously bind tumor-specific antigens and CD3^+^-positive cytotoxic T-cells in order to facilitate tumor cell destruction [84]. While BiTEs were first hypothesized in the 1960s and later constructed in the 1980s, it was in 2014 that the CD19-targeting blinatumomab became the first to receive FDA approval for the treatment of ALL, and to date, eight such therapies have been approved for oncologic indications [84]. In four major RCTs examining the use of blinatumomab for ALL, none have reported a toxicity or adverse event of DVT or PE, though the most recent, published in 2024, reported one thromboembolic event in each of the treatment (N = 78) and control groups (N = 70) [85,86,87,88]. Meanwhile, a single case of PE was reported in a phase I/II dose expansion trial of epcoritamab, a CD20-targeting BiTE approved in select cases of LBCL [89]. In a trial examining tebentafusp, a BiTE approved for metastatic uveal melanoma, two cases of PE were reported in the treatment group (1% of a treated N = 245) compared to three cases among controls (3% of 111) [90]. And in the case of tarlatamab, approved for small-cell lung cancer, a phase 2 trial reported three cases of PE (1.4% of a treated N of 220) [91]. In the remaining four FDA-approved BiTEs, VTE is not a reported toxicity; for glofitamab, a CD20-targeting BiTE approved for LBCL in 2023, neither two recent RCTs reported on adverse VTEs [92,93]. The same is true for talquetamab and teclistamab, each approved for multiple myeloma, based on RCTs conducted in the two agents separately and in combination, and in mosunetuzumab, approved for follicular lymphoma [94,95,96,97,98].

Importantly, a large pharmacovigilance analysis using the FDA Adverse Event Reporting System database from 2014–2023 revealed more concerning signals. Among 3668 BiTE-related adverse event reports, BiTEs as a class were significantly associated with disseminated intravascular coagulation, with a reporting odds ratio of 3.22 (95% CI 2.16–4.79) [99]. Blinatumomab was the primary driver of this association, and fatal cardiovascular events were also increased [99]. The true incidence of thrombotic complications with BiTEs likely remains underestimated, as most trials were not designed to systematically capture thrombotic events, and reporting has been inconsistent across studies.

### 5.2. Pathophysiology

BiTEs induce rapid T-cell activation upon binding both CD3 on T-cells and tumor antigens, triggering CRS [100]. This syndrome is characterized by massive release of pro-inflammatory cytokines including IFN-γ, TNF-α, IL-6, and others, typically peaking 2–5 days after treatment initiation [101,102]. These cytokines directly activate coagulation through multiple pathways already described, including the upregulation of TF expression on monocytes and endothelial cells (by TNF-α) as well as factor VIII and fibrinogen levels with concomitant anticoagulant protein suppression (IL-6) [56,57,103,104]. Additionally, BiTE-activated T-cells can directly damage endothelial cells through several mechanisms: activated T-cells release perforin and granzymes that may cause collateral endothelial injury, particularly in areas of high tumor burden or vascular tumor infiltration [84]. The endothelial dysfunction manifests as increased vascular permeability (contributing to capillary leak syndrome), upregulation of adhesion molecules, and shift toward a prothrombotic phenotype [100]. Effective BiTE therapy also induces tumor cell lysis, the extent of which correlates with toxicity, with higher tumor burden associated with more severe CRS and potentially greater thrombotic risk [101].

The strong association between BiTEs and DIC suggests activation of systemic coagulation with consumptive coagulopathy [99]. DIC has been specifically listed as a manifestation of severe CRS in blinatumomab prescribing information. The mechanism likely involves the convergence of multiple prothrombotic stimuli, including cytokine release en masse, widespread endothelial activation with TF upregulation, and release of procoagulant microparticles from activated T-cells and dying tumor cells.

## 6. Oncolytic Viral Therapy

### 6.1. Epidemiology

Data on thrombotic complications associated with oncolytic viral therapy remain limited, reflecting both the relatively recent clinical development of these agents and their current restricted indications. Talimogene laherparepvec (T-Vec), a genetically modified Herpes simplex virus, was the first FDA-approved oncolytic virus for cancer treatment, and provides the most comprehensive safety data currently available. In the pivotal phase III OPTiM trial comparing T-Vec to granulocyte-macrophage colony-stimulating factor (GM-CSF) in 436 patients with unresectable stage IIIB-IV melanoma, deep vein thrombosis was reported as a grade 3–4 adverse event in 1.7% of patients receiving T-Vec [105]. This rate is notably lower than the rates of ICI- or chemotherapy-associated thromboembolism synthesized in this review, though direct comparisons are limited by differences in patient populations and follow-up duration.

Clinical experience with other oncolytic viruses remains predominantly in early-phase trials with smaller patient cohorts. A systematic review of oncolytic virotherapy trials encompassing herpes simplex virus, coxsackievirus A21, adenovirus, poxvirus, and reovirus platforms found that serious adverse events were rare, with no specific signal for increased thrombotic risk [106]. However, these studies were not specifically powered to detect thrombotic complications, and standardized reporting of vascular events has been inconsistent across trials. Furthermore, most oncolytic viral therapies are administered intratumorally rather than systemically, which may theoretically reduce systemic prothrombotic effects compared to intravenous cancer therapies. The combination of oncolytic viruses with ICIs, currently under investigation in multiple trials, may alter this thrombotic risk profile. Of note however, the MASTERKEY-265 trial comparing T-Vec with pembrolizumab versus pembrolizumab alone for advanced melanoma did not report any thrombotic events among the 692 patients included [107].

### 6.2. Pathophysiology

While there is at present insufficient evidence to determine a comprehensive thromboembolic risk profile for oncolytic viruses, a number of hypotheses address the potential interplay between these emerging therapies and coagulation. For one, oncolytic viruses can directly impact tumor vasculature through invasion of tumor-associated endothelial cells: vesicular stomatitis virus, which demonstrates natural tropism for tumor blood vessels, is one such agent [108]. While this effect is therapeutically beneficial for tumor destruction, systemic vascular perturbations cannot be excluded. Second, oncolytic viral infection triggers robust inflammatory responses, characterized by the release of pro-inflammatory cytokines including TNF-α and IFN-γ, among others (T-Vec in particular has been engineered to express GM-CSF) [109]. As discussed earlier, such an inflammatory milieu may promote endothelial activation, increase TF expression, and enhance platelet reactivity. Third, viral-mediated tumor cell lysis releases damage-associated molecular patterns, including cell-free DNA and histones, which may activate coagulation through multiple pathways [110]. Finally, different viral platforms may carry unique prothrombotic potentials; vaccinia virus, for example, employed in oncolytic platforms such as JX-594, expresses viral proteins that can activate EGFR-RAS pathways, potentially affecting coagulation factor expression [111].

## 7. Risk Assessment and Prevention Strategies

The ability to accurately identify patients at highest risk for CAT is crucial for implementing targeted prevention strategies. Unlike traditional chemotherapy, where established risk factors and validated assessment tools guide prophylaxis decisions, the relatively recent widespread adoption of immunotherapy, combined with the dynamic nature of immune responses, further complicates risk assessment. Of seven published risk assessment models for CAT, including the Khorana score [112], the CATSCORE [113], the COMPASS-CAT score [114], the Tic-Onco score [115], and several modifications to the Khorana score including the 2010 Vienna modification [116], the 2012 PROTECHT modification [117], the 2013 CONKO modification [118], and the 2017 ONKOTEV modification [119], none include receipt of immunotherapy as a factor in their risk stratification model. However, more recent scoring models have included patients receiving immunotherapy in their original derivation or in subsequent validation studies, and at least one (the Khorana score, Table 1) has been validated for risk stratifying patients receiving ICI therapy [112,120,121]. However, there is also evidence to suggest the utility of the Khorana score may be limited in this population, with a 2024 study finding ICI recipients with lung cancer described most (N = 7) VTE events occurring in a low risk-stratified group [122]. Also, in 2024, Liang et al. published the first validated nomogram model specifically for predicting VTE risk in lung cancer patients treated with ICIs [123]. This comprehensive model incorporated ten predictor variables and demonstrated superior discriminatory ability compared to the Khorana score in the authors’ training and validation datasets [123].

To the best of our knowledge, no trial has been published assessing the role of targeted thromboprophylaxis in patients receiving immunotherapy. The 2021 ASCO irAE management guideline update addresses elevated VTE risk among ICI recipients, and notes management is directed at treating and preventing VTE complications, avoiding immunosuppressive therapy and, once a patient’s condition has stabilized, to resume ICI therapy in the absence of other irAEs [124].

## 8. Future Directions and Unresolved Issues

The rapidly expanding landscape of cancer immunotherapy has introduced new complexity to the management of CAT. As detailed in this review, novel immunotherapeutic agents, including ICIs and CAR T-cell therapies among others, have demonstrated substantial rates of VTE. Despite growing recognition of these risks, critical knowledge gaps remain. The pathophysiological mechanisms linking immune activation to thrombosis remain only partially understood. Pre-existing risk stratification models have not been comprehensively tested nor do guidelines specifically address the prevention of CAT in immunotherapy recipients. Further, there is no consensus that VTE management in the context of immunotherapy should be treated differently [125,126,127,128].

### 8.1. Emerging Immunotherapies for Cancer

Breakthrough forms of adoptive cell therapy, such as TILs and CAR-NK cells, as well as a host of new ICI-targeting antibodies (such as the inhibitory immunoreceptors lymphocyte activation gene 3 and the transmembrane protein TIM3, which function similarly to PD-1 and CTLA-4), represent a small selection of emerging immunotherapies under trial, each with a distinct mechanisms of action and incompletely understood thrombotic potential [72,129,130]. Understanding the mechanisms linking immune activation to thrombosis remains a critical research priority.

### 8.2. Personalized Prophylaxis and Treatment Strategies

As discussed earlier, there are limited tools for VTE risk assessment among immunotherapy recipients, with even the most widely-tested scoring system performing inconclusively among ICI recipients, one of the most widely eligible forms of cancer immunotherapy [122]. Future research should focus on developing immunotherapy-specific risk assessment models, potentially incorporating biomarkers which prospective investigation has been associated with an elevated risk of CAT, both venous and arterial. The development of personalized thromboprophylactic strategies must also account for unique bleeding risks in certain immunotherapies, particularly CAR T-cell therapy, where treatment related thrombocytopenia is common [16,64].

### 8.3. Real-World Evidence

The incidence of VTE in RCTs has been described as highly underreported, and the discrepancy between lower VTE rates reported in clinical trials versus substantially higher rates in real-world cohorts highlights the need for prospective registries designed to capture the true incidence of thrombotic events [131]. These registries should include standardized definitions and, where possible, systematic screening protocols, as well as long-term follow-up.

## 9. Conclusions

The advent of cancer immunotherapy has revolutionized oncology treatment but has also brought with it challenges, such as the risk of CAT. This review highlights the burden of VTE in patients receiving immunotherapies such as ICIs and CAR T-cell therapies. While our understanding of the pathophysiological mechanisms involved remains incomplete, emerging evidence suggests that immune activation plays a central role in promoting hypercoagulability.

Current risk assessment tools developed for chemotherapy-treated patients may be insufficient to adequately characterize the risk of immunotherapy-associated VTE, necessitating the development of novel prediction models. The management of VTE follows general cancer thrombosis guidelines, though specific considerations, such as bleeding risk among CAR T-cell therapy recipients, remain essential.

The changing landscape of cancer and cancer treatment represents a significant hurdle to overcoming the problem of VTE in immunotherapy. Increasingly heterogenous immunotherapeutic agents, combination regimens and patient populations, as well as evolving standards of care, pose a challenge to investigators. Dedicated research efforts are urgently needed to optimize thromboprophylaxis, develop better tools for risk prediction, and understand the mechanistic underpinnings of immunotherapy-associated thrombosis. Only through such comprehensive efforts can the burden of thrombosis be minimized while maximizing the therapeutic benefits of these revolutionary treatments for cancer.

## Figures and Tables

**Table 1:** The Khorana Risk Score for Venous Thromboembolism in Cancer Patients.

Patient Characteristics	Score
Cancer site	
Very high risk (stomach, pancreas)	+2
High risk (lung, lymphoma, gynecologic, bladder, testicular)	+1
Pre-chemotherapy laboratory values	
Platelet count 350 × 10^9^/L	+1
Hemoglobin < 10 g/dL (or use of red cell growth factors)	+1
Pre-chemotherapy leukocyte count > 11 × 10^9^/L	+1
BMI	
BMI ≥ 35 kg/m^2^	+1
**Risk categories**	**Score range**
Low risk	0
Intermediate risk	1–2
High risk	3–6

The Khorana score for ambulatory risk assessment of chemotherapy-associated thromboembolism. Adapted from reference [112].

## Abbreviations

The following abbreviations are used in this manuscript:

ALL	acute lymphoblastic leukemia
BiTE	bispecific T-cell engager
CAR	chimeric antigen receptor
CAT	cancer associated thromboembolism
CRS	cytokine release syndrome
CTLA-4	cytotoxic T-lymphocyte associated protein 4
DOAC	direct oral anticoagulant
DVT	deep vein thrombosis
FDA	Food and Drug Administration
GM-CSF	granulocyte-macrophage colony-stimulating factor
HR	hazard ratio
ICI	immune checkpoint inhibitor
IEC-HS	immune effector cell-associated hemophagocytic lymphohistiocytosis-like syndrome
IFN-γ	interferon-γ
IL	interleukin
LBCL	large B-cell lymphoma
MDSC	myeloid-derived suppressor cells
NET	neutrophil extracellular trap
PE	pulmonary embolism
PD-1	programmed cell death protein 1
RCT	randomized controlled trial
sVCAM-1	soluble vascular cell adhesion molecule 1
TCR	T-cell receptor
TF	tissue factor
TIL	tumor infiltrating lymphocyte
TNF-α	tumor necrosis factor α
VTE	venous thromboembolism
